# Cost-Effective Recruitment Strategies That Attract Underrepresented Minority Undergraduates Who Persist to STEM Doctorates

**DOI:** 10.1177/2158244016657143

**Published:** 2016-07-15

**Authors:** Cherilynn R. Shadding, Dawayne Whittington, Latricia E. Wallace, Wambul S. Wandu, Richard K. Wilson

**Affiliations:** 1 Washington University School of Medicine in St. Louis, MO, USA; 2 Strategic Evaluations, Inc., Durham, NC, USA; 3 National Eye Institute, Bethesda, NIH, MD, USA

**Keywords:** recruitment, underrepresented minority, cost-effective, summer programs, undergraduate research, STEM

## Abstract

The paucity of underrepresented minorities (URMs) earning science, technology, engineering, and mathematics (STEM) degrees remains an issue in revitalizing the U.S. biomedical workforce. Due to reductions in federal funding, maintaining the integrity of programs that focus on URM retention and recruitment is crucial. We present data on the mechanisms used to recruit URM students to our program (e.g., email, events, referrals, website), which individually were equally effective in attracting applicants to the program. Recruitment mechanisms were grouped and further classified relative to their cost to implement as lower and higher cost. Our results indicate that lower cost mechanisms, statistically, were as effective as higher cost mechanisms in recruiting students who persisted to PhD programs. Using a binary logistic regression model to predict PhD matriculation, higher cost mechanisms were not significant predictors of PhD matriculation. Collectively, these data demonstrate for the first time that lower cost mechanisms can be as successful in recruiting URM students to summer programs who pursue PhDs in STEM fields.

## Introduction

The fields of science, technology, engineering, and mathematics (STEM) represent areas of economic growth and prosperity for the United States. However, STEM fields also indicate the social ills that remain in U.S. society. Racial, ethnic, and gender disparities in STEM education and degree attainment have been documented for nearly 40 years and are not likely to disappear in the near future (George, Neale, Van Horne, & Malcom, 2001; National Academy of Sciences, 2010). According to data from the National Science Foundation (NSF; 2013; 2002–2012), underrepresented minorities (URMs), although representing 32% of the U.S. population, comprise only small percentages of bachelor’s degrees earned in science and engineering fields (U.S. Census Bureau, 2015). Specifically, Hispanics (all races) comprise 9%, Blacks/African Americans 9%, and American Indian/Native Americans less than 1%. With regard to doctoral degrees, only 11% are earned by URM and if behavioral and social sciences are ignored, this drops to a striking 9%. This disparity in degree attainment directly translates to a non-diverse STEM workforce of nearly five million, with one study reporting that URM represent less than 9% of the workforce and account for less than 8% of the doctorate-requiring positions in STEM universities and 4-year institutions (Poirier, Tanebaum, Storey, Kirshstein, & Rodriguez, 2009). NSF (2013) data from 2010 show that URM comprise about 13% of the STEM workforce at the bachelor’s degree and higher, while less than 6% hold full professorships across all U.S. universities.

To address this education and workforce gap, many programs have been established to enhance recruitment and retention of URM in STEM, with agencies such as the National Institutes of Health (NIH), the NSF, and other government agencies serving as large benefactors. These programs vary, from summer and academic year research experiences to innovative STEM curriculum in the classroom (Chaplin, Manske, & Cuise, 1998; Mervis, 2010; National Research Council [NRC], 2005; Schultz et al., 2011). They have demonstrated that research participation is a key element to career intentions and decisions and that improvement in curriculum aids in retention of STEM majors. Whether solely dedicated to URM or a commitment to URM, the focus of such programs is improvement in diversity in STEM.

In order for the United States to maintain its competitive economy and world standing in technology and innovation, the federal government and others have been implored to target URM as an “untapped” resource (George et al., 2001; National Academy of Sciences, 2007; Palmer, Maramba, & Dancy, 2011; Rodriguez et al., 2012) However, perturbations in federal funding in recent years have affected these diversity efforts that have been ongoing for several decades. The effects of sequestration reduced the budgets of NSF and the NIH by 5%, and have just rebounded, with NIH receiving its highest increase in 12 years in the new Bipartisan Budget Act of 2015 (“Bipartisan Budget Act of 2015,” 2015; “President Obama Inks Budget,” 2015; Wadman, 2013). In spite of this increase, funding for diversity research programs represents a mere fraction of the budgets for NIH and NSF. Institutional and individual training grants, research centers at minority institutions, and minority biomedical research support, which are the likely NIH funding mechanism for diversity research programs, collectively represent 3% of the 2016 NIH budget, which includes programs not directed to diversity (NIH Office of Budget, 2015). For NSF, the education programs represent 12% of the 2016 budget, but again these activities are not solely focused on diversity (NSF, 2015). This paucity of funding presents a challenge to staff and program directors of diversity research programs. One area of specific challenge is student recruitment. Federally funded programs (e.g., NIH Education Projects [R25s]) provide limited resources for student recruitment and in some cases are not allowed, resulting in the need for program directors to find other resources for recruitment (e.g., university support). For programs where recruitment strategies are vital, such as summer research experiences, developing and utilizing efficient recruitment strategies may be necessary to sustain the quality and impact of these programs.

## Literature Review

Research opportunities for undergraduates, including summer research programs, have shown positive effects on retaining students in STEM (Barlow & Villarejo, 2004; Pender, Marcotte, Sto Domingo, & Maton, 2010; Slovacek, Whittinghill, Flenoury, & Wiseman, 2012). The benefits of undergraduate research to all students are well known and include higher interest in science, developing identity as a scientist, improved self-confidence, research and professional skill development, confirmation of career path, and learning gains in the research process, lab techniques, and scientific problem solving (Junge, Quinones, Kakietek, Teodarescu, & Marsteller, 2010; Kardash, 2000; Lopatto, 2004; Seymour, Hunter, Laursen, & Deantoni, 2004). Programs directed to URM have shown a great impact on career trajectories, college persistence, undergraduate grade point average (GPA), and earning a biology degree (Matsui, Liu, & Kane, 2003; Nagda, Gregerman, Jonides, von Hippel, & Lerner, 1998; Villarejo, Barlow, Kogan, Veazey, & Sweeney, 2008). Other programs, including the well-known Meyerhoff Scholars Program, have been shown to add to the scientific workforce by producing URM who persist to PhDs in STEM (Maton, Sto Domingo, Stolle-McAllister, Zimmerman, & Hrabowski, 2009). One common strand for these studies is evaluation and a rigorous analysis of the effect of the programs on the aforementioned outcomes, representing a cadre of literature that further validates the necessity of undergraduate research. Furthermore, these studies are a mixture of summer and academic programs for which some extensive recruitment mechanisms may not have been necessary. While recruitment is mentioned in some of these studies, limited data are presented regarding how students were recruited to these programs. Investigations on the correlation of recruitment methods to participant outcomes are lacking in the literature.

Recruitment is a key component for summer research programs, especially for programs directed to target populations, where access to such populations may be limited locally. When the focus is narrowed to summer programs, there are few data reported on recruitment mechanisms. Data from the “Spend a Summer With a Scientist” program, directed to minority students, showed that 62% of the summer students who were surveyed entered graduate school, but there was no mention of how students were recruited (Alexander, Foertsch, & Daffinrud, 1998; Alexander, Foertsch, Daffinrud, & Tapia, 2000). Similarly, an evaluation of the Summer Undergraduate Research Experience (SURE) program at Emory University reports successful outcomes such as increased GPA and number of science courses taken postparticipation, but the only data related to recruitment showed that over 20% of students were from liberal arts colleges and that 75% of non-Emory students were from out of state (Junge et al., 2010). In a study of the Research Experiences for Undergraduates (REUs) program sponsored by the NSF, principal investigators across 106 REU sites were asked to report measures of success of the participants, which included graduate school attendance, participant co-authorship, and participant satisfaction (Beninson, Koski, Villa, Faram, & O’Connor, 2011). The mechanisms used to recruit were Internet (email, website), mailings, media, conferences, campus recruitment office, and other methods, with the majority of participants recruited by Internet or conferences. Although graduate school attendance was a marker of success, the study did not indicate the number or percentage of students in graduate school, thus any link of outcome to recruitment mechanism could not be assessed. An evaluation report of the programs funded by the Biological Sciences Directorate of NSF (BIO REUs) indicates gains by participants in research skills and thinking like a scientist, with over 50% intending to pursue a PhD in a STEM field. The recruitment mechanisms utilized were announcements, the NSF website, academic advisors, and web searches. An updated report from this group discussed the types of institutions of participants: over 70% from Research I and over 84% from public universities, but neither institution type nor mechanism type was linked to outcomes or other variables analyzed in the report (Weston, 2012, 2013). An earlier evaluation of a summer research program targeted at URM across 15 R1 institutions within a Midwest consortium showed that 75% of participants went on to graduate or professional school, where these students were a mixture of majors (e.g., humanities, engineering, natural, and physical sciences; Foertsch, Alexander, & Penberthy, 1997). Students were recruited to the program from “on campus” or “off campus” of the host institution. The “off campus” mechanisms included partnerships with minority-serving institutions (MSIs), which represented 9% of the participants. Their data showed that participants who were STEM majors were more likely to attend graduate school and to be recruited from “off campus”–research extensive schools. No further classifications of recruitment methods were mentioned in the report.

From these studies, it is evident that resources have been devoted to increasing the pool of URM who will diversify the STEM workforce. However, little has been reported in the literature on how students were recruited. Missing in the literature is a practical focus on recruitment of URM students, especially to summer programs. To our knowledge, the literature to date has not demonstrated the success of recruitment in attracting students who eventually enroll in PhD programs and other STEM-related degree programs. As federal agencies provide limited funds for recruitment to diversity research programs, it is imperative for programs to enhance cost-effectiveness to continue the strides made in diversity.

Using data from our summer program targeted at URM, Opportunities in Genomics Research (OGR): Undergraduate Scholars, we show the outcomes of the participants and the recruitment mechanisms used to attract students to the program. We demonstrate empirically, we believe for the first time, the effectiveness of low-cost strategies in attracting students who are on the path to high impact terminal degrees in STEM, namely, STEM doctorates, to a summer program.

## Method

### Research Questions

**Research Question 1:** Are low-cost recruitment strategies effective in recruiting students to the OGR program?
**Research Question 2:** Are low-cost strategies effective in recruiting students who persist to PhDs in STEM?

### Program Design and Components

The OGR Program was established in 2007 at The McDonnell Genome Institute at Washington University in St. Louis. This program is a part of the Diversity Action Plan (DAP), an initiative by the National Human Genome Research Institute (NHGRI), to increase diversity in the field of genomics and related fields. The goal of the DAP programs is to increase the number of PhDs in the field from underrepresented backgrounds. The DAP programs initially defined URMs as African American, Hispanic, Native American, and Pacific Islander. Since 2011, it has expanded the definition to include first-generation college attendees, socioeconomically/culturally disadvantaged, and persons with learning and physical disabilities.

Here we report on the summer research program of OGR: Undergraduate Scholars. Undergraduate Scholars is an 8-week summer program where students conduct research with Washington University faculty whose research focus is concentrated on or related to genetics or genomics. The program consists of activities to encourage careers in research and to help prepare students for graduate school and its application process. Such activities include graduate school preparation, Graduate Record Examination (GRE) preparation, seminars, journal clubs, scientific presentation skills, and career planning. The foci of this article are the recruitment mechanisms and potential links to outcomes, rather than the program features.

### Participants

We report on 185 applicants to the OGR program from 2007 to 2013. Participants were selected to OGR based on their application and phone interviews conducted by program staff. Factors influencing acceptance into OGR included recommendations by the mentors, GPA (overall and STEM), undergraduate major, research interests and experience (for upperclassmen), interest in a PhD or research career as indicated in a personal statement and interview, and classification at time of application. Eligible students were freshmen, sophomores, juniors, or first semester seniors (students scheduled to graduate the winter following the summer program).

We note we did not use a hard-line GPA in our selection process although great consideration was given to GPAs 3.3 and above. Using the NSF definition of STEM (science and engineering), 100% of participants (*n* = 59) were STEM majors upon entry to OGR, including two behavioral science majors. Of the program alumni (*n* = 55), from 2007 to 2013, 98% obtained a bachelor’s degree in STEM.

### Data Collection

The baseline data were extracted from applications to the program and internally stored in Microsoft Excel. OGR students’ long-term career outcomes, including career path data, were collected from participants and stored in iBioSketch. com, an Internet-based career tracking tool designed by Strategic Evaluations, Inc., our external evaluation team. These career outcomes were verified through at least two other sources, including (a) study leaders’ follow-up communication with alumni and their research mentors and (b) queries submitted to the National Students Clearinghouse. Matriculants were asked to initiate and update their profiles in iBiosketch annually, while formal surveys were given annually to biannually. We supplemented this information with informal tracking methods (phone calls, social media, emails, etc.). The reported outcome data indicate where students were in their career to our knowledge as of September 2014 or the last reporting of the student (e.g., PhD program, working in STEM field, etc.)

All data are reported in aggregate. Demographic information such as gender, race, and ethnicity were voluntary and self-reported on the OGR application. Required information included name of institutions, overall, and STEM GPA (verified by transcript). Institutions were classified as an MSI based on data from the Department of Education listings of minority institutions and Excelencia in Education (2014, Edexcelencia.org). The following categories for MSIs were used: Historically Black Colleges and Universities (HBCUs), Hispanic Serving Institutions (HSIs), and other MSIs (Tribal Colleges and Universities, Primarily Black Institutions, Native American–Serving Non-Tribal Institutions, Asian American, and Native American Pacific Islander–Serving Institutions), or non-MSIs (majority institutions or primarily White institutions). Institutions were further categorized using basic Carnegie Classification (Carnegie Foundation for the Advancement of Teaching, 2012) and condensed into the following major categories: associates (includes private and public), bachelor’s (includes baccalaureate arts and science, diverse, and baccalaureate/associates), master’s (small, medium, and large), doctoral (doctoral/research universities), and research (research university with high or very high activity).

### Recruitment Mechanisms

The main goal of this study was to investigate a link between recruitment methods and student outcomes, particularly low-cost recruitment methods. We have actively recruited for the OGR program and documented the mechanism of student recruitment. We provided several closed-ended options for applicants to indicate on the OGR application, “how they heard about the program,” which included instructor, program, email, conference, career fair, brochure/flyer, website, word of mouth, former OGR student, and other; applicants were asked to indicate details (e.g., conference name). These options were condensed into five main mechanisms: emails, referrals, events, websites, and other. A brief description of each method is found in Table 1. A recruitment database was maintained and included all individuals and institutions we contacted through emails, events, and mailings. A mechanism was indicated for an applicant as the likely initial mode of contact. For example, if a student listed instructor on his or her application, and it was noted in our recruitment database that several emails were sent to that applicant’s institution, then email was listed as the recruitment mechanism. If an applicant’s choice of recruitment mechanism could not be verified in our records, the strategy was listed as indicated by the applicant or, if no strategy could be verified, the mechanism was designated as “other” (see Table 1). The responses for each mechanism were tallied and the mechanisms assessed against demographics, GPA, institution types, and outcomes.

### Statistical Analysis

IBM SPSS Statistics Version 21 was used to compute descriptive statistics, as well as test for correlations and statistical significance. Independent-samples chi-square tests were used to test the distributions for categorical response variables, including differences between demographic variables, recruitment mechanisms, and career outcomes. When appropriate, the crosstab function within SPSS was used to help determine whether column proportions were significantly different. In these cases, *z* values were computed and *p* values were adjusted for multiple comparisons using the Bonferroni method. To determine whether the means for our scale variables (overall GPAs and STEM GPAs) were equal across our independent categories, one-way ANOVAs were conducted. Matriculation status and recruitment mechanism were the independent variables, while GPA and STEM GPA were the dependent variables. Finally, variables in the data set were examined using a logistic regression to determine which most likely explained students’ career outcomes. The eligible variables for consideration in the model were GPA, ethnicity, race, NSF quartile of funding, NIH quartile of funding, MSI classification, Carnegie classification, cost of recruitment mechanism, level of personal contact, and type of recruitment mechanism. Using this technique, five explanatory/predictor variables and one response variable (career outcome) were chosen for the logistic regression model. The dependent variable was defined as the degree pursuit of the former scholars of the program coded with dummy value: 1 = *scholars who chose to pursue a PhD* or 0 = *scholars who did not pursue a PhD*. Predictor variables used were coded as gender (1 = *female*, 0 = *male*), ethnicity (1 = *Hispanic*, 0 = *non-Hispanic*), Carnegie classification (1 = *high or very high research activity*, 0 = *non-research classified institutions*), recruitment mechanism (1 = *high-cost events and website*, 0 = *low-cost email and referrals*). The median and mean GPA were calculated to both be 3.5. Therefore, scholars with GPAs of 3.5 or higher were coded as 1, while those with GPAs lower than 3.5 were coded as 0. The logistic regression model had a Nagelkerke *R*^2^ value of .291, suggesting that nearly 30% of the variability in what predicted students pursuing PhDs was accounted for in this model.

## Results

### OGR Demographics

The OGR programs are directed to increasing the number of underrepresented students who pursue PhDs in genomics and related fields. Over the 7-year reporting period, this study demonstrates that the program expanded its reach to help accomplish this goal. In the first three tables, non-matriculant applicants (*n* = 126) are compared with matriculant applicants (*n* = 59), where a matriculant is defined as an applicant who was accepted and participated in the program.

Males and females were recruited to the program at rates that were not significantly different (χ^2^ = 2.31, *df* = 1, *p* = .128). However, the number and percentage of females in both the non-matriculant (66%; *n* = 83) and matriculant pools (54%; *n* = 32) trended higher than that of males. The percentage of females was nearly double that of males in the non-matriculant pool but was only 8% higher in the matriculant pool (Table 2). This may be reflective of gains made by women in undergraduate and graduate programs in certain sectors of STEM, namely, biological sciences, where females earn 60% of bachelor’s and 56% of doctorates in this area but 51% of bachelor’s and 46% of doctorates in STEM overall (Burrelli, 2008; NSF, 2013). These data may be indicative of another trend where minority males lag behind their female counterparts in certain areas of STEM, where Black and Hispanic men earn 36% and 44% of STEM bachelor’s and 40% and 46% of STEM doctorates, respectively (Aud, Fox, & KewalRamani, 2010; Harper, 2012; NSF, 2013) A more rigorous analysis would be needed to make this determination. In light of this potential disparity, our recruiting reflects an effort to increase male matriculants. In 2007, we only had one male student in our program, constituting 13% of program participants in that year. Over time, males represented a higher percentage of the matriculants, peaking at 75% in 2009 (data not shown).

On the OGR applications, information on ethnicity and race was requested. Using the census classification for ethnicity and race, the number and percentage of Hispanics and non-Hispanics (all races) and the number and percentage of racial groups (including Hispanic ethnicity) are reported (Table 2). Using federal classifications, persons choose an ethnicity and a race; thus, a person who self-identifies as Hispanic (ethnicity) may also identify as Caucasian (race). Comparing non-matriculants and matriculants, Hispanics were selected at a higher rate than non-Hispanics for matriculation in the program. Forty-four percent of the matriculants identified as Hispanic; however, they only constituted 31% of the applicant pool (58 out of 185 applicants; χ^2^ = 5.47, *df* = 1, *p* = .019). Blacks/African Americans constituted 41% of non-matriculants and 49% of matriculants. As Hispanics are included in the race category as well, they are reflected in some of the racial groups. However, the majority of Hispanic applicants chose “no response” to race on the application, *n* = 44. Caucasians represented a small portion of the applicant pool at 16%, where 21 of 27 applicants and two of three matriculants identifying as Caucasian were non-Hispanic Whites (χ^2^ = 13.802, *df* = 5, *p* = .017). Six applicants chose neither an ethnicity nor race; thus, race and ethnicity were recorded for 120 individuals in non-matriculants.

### Selectivity

**Figure 1.**
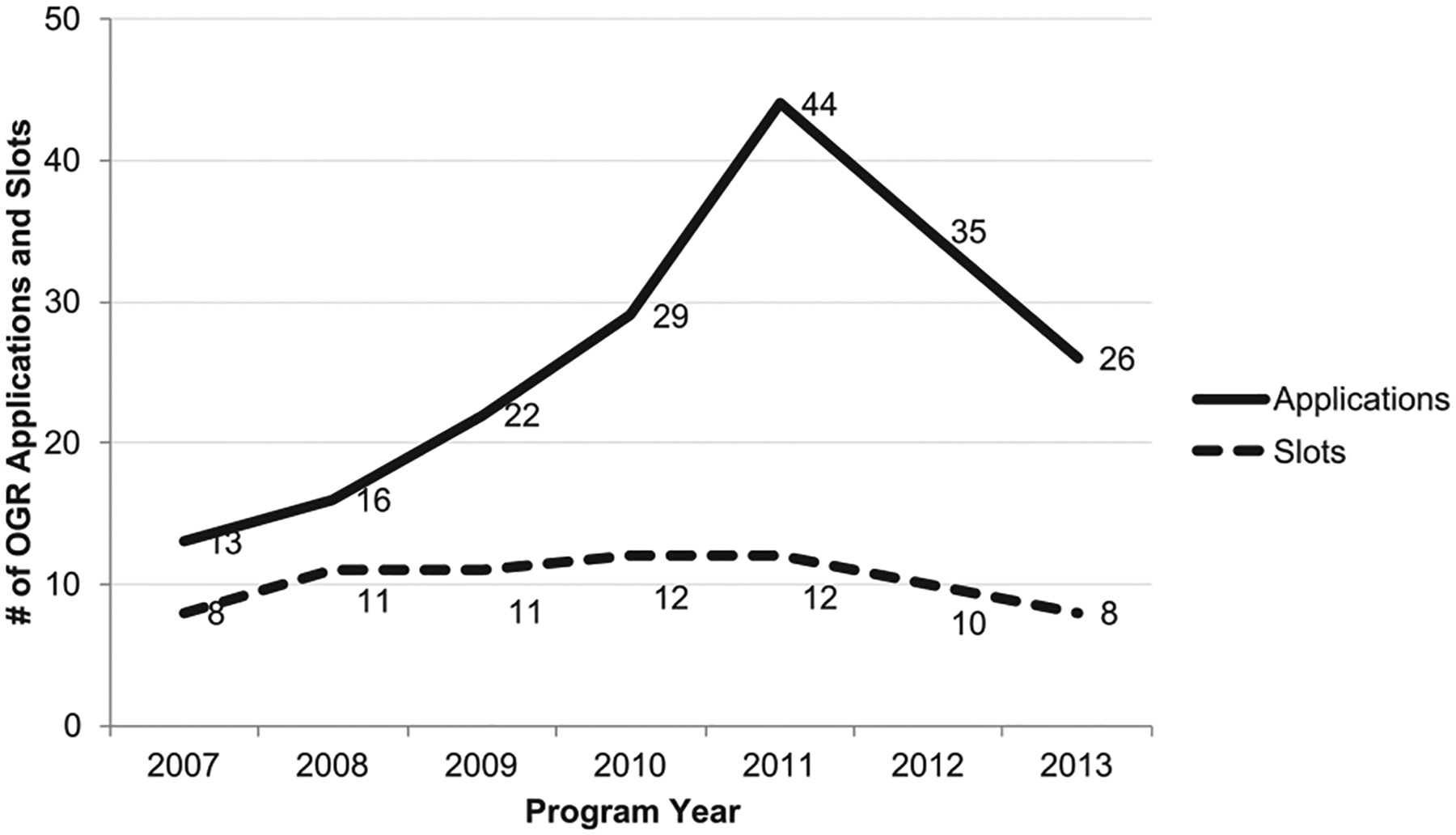
Comparison of the number of OGR applications (*n* = 185; solid line) and program slots (*n* = 72; dashed line) disaggregated by program year from 2007 to 2013. *Note*. The overall acceptance rate for the 7-year period was 33%, where the total matriculants (*n*) = 59. OGR = Opportunities in Genomics Research.

The OGR Undergraduate Scholars program has remained very small, with eight to 12 participants per year. Initially, recruitment efforts were limited but have diversified over the years of the program. Since its inception, the number of applicants to the program increased over 300% at its peak in 2011, with an overall acceptance rate of 33% from 2007 to 2013 (59/185). While the total number of applicants declined from 2012 to 2013, the acceptance rate was maintained close to 25% due to fewer available slots. The OGR program participants included previous students in good standing who re-entered the program non-competitively (Figure 1), however, these students are only counted once in the total number of applications, so each year reflects unique applicants only (solid line—Figure 1).

To better assess demographics and the selectivity of OGR, institutional data were extracted from the applications. Institutions were classified as MSI using data from the U.S. Department of Education minority institutions programs and Excelencia in Education. The major classifications utilized for MSIs were HBCU, HSI, other MSI, or non-MSI (Table 3). For non-matriculants, 50% had attended an MSI, and 26% had attended an HBCU. Among matriculants, over 60% attended an MSI where HBCUs and HSIs were nearly equal at 31% and 32%, respectively; these differences were not significant. In addition, Carnegie classifications for matriculants and non-matriculants were compared and condensed into the following categories: associates, baccalaureate, master’s, doctoral, and research. Research universities provided the largest number of applicants, both matriculants (44%) and non-matriculants (39%; Table 3). Baccalaureate and master’s institutions supplied the next largest group of non-matriculants (25% and 28%) and matriculants (27% and 17%), respectively. Overall, there was no difference in matriculants compared with non-matriculants when considering Carnegie classification. Regional data for the institutions using U.S. regions as classified by U.S. Census Bureau were collected. The largest percentage of schools for both matriculants (53%) and non-matriculants (38%) were located in the South, which corresponds to the location of a considerable number of MSIs (data not shown)

The selectivity of the program is further demonstrated by the GPA data. To determine whether there was a difference in GPA within different phases of the application process, we analyzed GPAs of all applicants, those invited to interview, offers made, and matriculants. We found no significant difference in overall or STEM GPA among applicants within the different phases (data not shown). Therefore, GPA variables were not distinguishing factors between students at different phases of the application process.

### Recruitment Mechanisms

Suggested best practices to recruit and retain URM in STEM include strategies such as improving K-12 education, partnerships with MSIs and secondary schools, establishing mentorship programs, discipline-specific short courses, and so on (NHGRI, 2006; Organization for Tropical Studies, 2007). Little attention has been paid to the practical aspect of recruitment, which may become more important for federally funded programs given the decreases recently experienced in funding.

Table 4 shows recruitment mechanisms for non-matriculants and matriculants to the OGR program from 2007 to 2013. For both groups, the largest percentage of students was recruited via email and referrals; the number recruited from emails trended slightly higher for non-matriculants and referrals for matriculants. Collectively, the two strategies of referrals and emails accounted for nearly 70% of students who ultimately were selected for the program. However, when tested statistically, all recruitment strategies proved to be equally effective in drawing students to the program. The lack of significant difference also indicates that no specific mechanism was favored in the selection of students. These data provide some confidence that the methods were varied and provide a platform to effectively compare them for outcomes.

In this study, with the exception of ethnicity and race, there was no significant difference in non-matriculants versus matriculants in other demographics, institution classification, GPA, and recruitment mechanism. Next, data were analyzed to determine whether there was a difference in these variables when measured against the recruitment mechanism.

Table 5 shows the recruitment mechanism of all applicants by gender, ethnicity, and race. Crosstabs of demographic variables and recruitment mechanisms show that there were notable gender differences. Males were more likely to apply to the OGR program as a result of a referral (44%) or through website advertisement (16%). Conversely, females were more likely to apply to the OGR program as a result of email solicitations (40%) or by hearing about the OGR program at an event (30%). Therefore, there may be gender differences in how applicants were attracted to the program.

While there was no significant difference across any of the mechanisms when comparing ethnicity, the effectiveness of recruitment mechanisms varied significantly for the different racial groups. Applicants identifying as Black/African American were most likely drawn to OGR by interacting with our program at an event (31%). Website advertisements were the most effective mechanisms for attracting applicants identifying as Asian, where 33% applied through this mechanism, more than any other racial group. Email solicitations attracted a significantly high proportion of applications from students identifying as Caucasian (50%).

Our recruitment mechanisms, emails, events, referrals, website, and other, experienced varying levels of success dependent upon the type of institution (Table 6). Students attending institutions classified as HBCUs were more likely drawn to the program through interacting with OGR project leaders at an event (41%), but website advertisement was most effective in drawing applicants from non-MSI institutions (15%). The recruitment mechanisms were equally as effective across the different Carnegie classifications although emails or referrals were the mechanisms that attracted the largest percentage of applicants from each institution type.

We also tested whether any mechanism was responsible for attracting applicants with more selective overall or STEM GPAs. While the overall and STEM GPA for applicants recruited through the website mechanism trended higher than the GPA data for applicants recruited through other mechanisms, there was no statistical difference indicating that no given strategy was more effective in attracting a student with a high GPA (data not shown).

### Outcomes

The recruitment mechanisms utilized in this study are relatively common and accessible for similar programs. The goal of this study was to demonstrate, for the first time, the effectiveness of these methods in recruiting students who progress to desired program outcomes.

Table 7 lists the outcomes of the students in the OGR program from 2007 to 2013. Only students who completed their baccalaureate degree are included in the table (55 students).

Program outcome data reveal that 27% of students entered PhD programs (includes MD/PhD, *n* = 1) while 16% transitioned to professional degree programs in STEM areas (MD, DDS, and PharmD). An additional 11% enrolled in a postbaccalaureate training or master’s degree programs following their undergraduate studies. Collectively, 54% of students across the 7 years transitioned to advanced training in STEM fields. It is important to note that the “outcomes” used in this analysis were accurate as of September 2014; students may have progressed to a higher level of training since this report. These data are a snapshot of where students were at the time our team analyzed the data for the article and does not capture each career step made by the participant since completing OGR.

An analysis of demographic variables in relation to career outcomes shows that there were no statistical differences for the gender variable. Fifty-three percent of the students enrolled in PhD programs were male, while 67% of students enrolled in STEM professional degree programs were female (Table 8).

Hispanics showed a strong trend in transitioning to PhD programs (67%) versus just 33% of non-Hispanics. However, the difference was not significant (χ^2^ = 5.031, *df* = 3, *p* = .170). No statistical differences were seen among career outcomes and race. While Black/African American students accounted for more than 50% of students transitioning to STEM professional degree programs and postbaccalaureate/master’s programs, these students accounted for only 26% of those transitioning to PhD programs.

Students who attended institutions categorized by Carnegie as research high/very high were more likely to enroll in a PhD program than to pursue other career paths (Table 9). Seventy-three percent of students who enrolled in PhD programs were trained at these institutions, a higher percentage than any other Carnegie classification. There were no additional differences found by analyzing Carnegie type versus career outcomes.

It is interesting to note that 40% of students trained at HSIs went on to pursue a PhD, outpacing their HBCU counterparts nearly threefold. However, the difference in PhD enrollment rates between HSIs and HBCUs were not significantly different (χ^2^ = 3.760, *df* = 6, *p* = .709).

When comparing outcomes versus the mechanism of recruitment, the majority of students who transitioned to PhD programs were recruited by events (40%), while the majority of students who transitioned to STEM professional degree programs were recruited by referrals (55.6%; Table 10). However, there were no statistically significant differences in the individual recruitment mechanisms that drew students to the program and their ultimate career outcomes, suggesting that no single mechanism was statistically more likely to yield students enrolling in advanced degree programs, *F*(4, 50) = .858, *p* = .496. Using η^2^ as the measure of effect size, recruitment mechanism only accounted for 6% of the total variability in the career outcomes.

**Figure 2.**
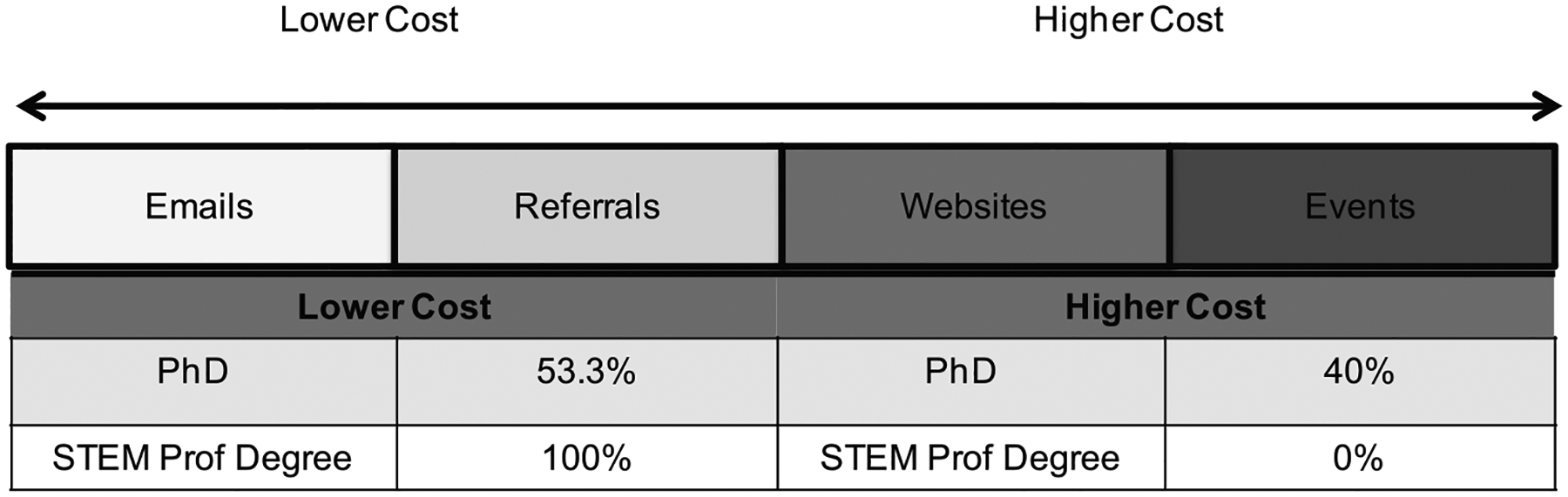
Terminal degree outcomes for OGR students completing an undergraduate degree 2007–2013 (n = 55) disaggregated by relative cost of recruitment mechanism. Lower cost mechanisms = emails and referrals; higher cost mechanisms = websites and events. The gradations of color represent the relative increase in cost of the mechanism. Percentages are the students who pursued a PhD or a STEM professional degree (MD, DDS, or PharmD). *Note. F*(1, 53) = 1.172, *p* = .284; observed power = .186, η^2^ = 0.02 (PhD pursuit vs. Cost).

This study aimed to determine the effectiveness of low-cost recruitment strategies in attracting students who later enroll in PhD programs. To further explore these mechanisms and their outcomes, strategies were grouped by relative cost: lower cost and higher cost. Figure 2 shows a relative cost spectrum of the recruitment mechanisms we used. Emails and referrals were of little cost in dollars to the OGR program to execute as a mechanism; thus, we combined these to form the lower cost category. However, websites and events are higher cost strategies. Website here includes our website as well as other websites across the country where we advertised (e.g., schools, government). Although we advertised for free on external sites, generally speaking websites can be costly to create and to maintain, where a basic site can be free or cost several thousand dollars and more elaborate sites can be as high as tens of thousands (Katkin, 2015). Events can be costly for small programs, whereas for our program, they have been primarily conferences, ranging in cost from US$1,500 to US$3,000 or more per conference. In Figure 2, we used the two-directional arrow for events and websites to indicate that one may cost more than the other, but both are more costly than emails or referrals for our program.

Lower cost mechanisms were highly effective in attracting students who eventually matriculated into terminal degree programs. Over 50% of OGR alumni who pursued PhD programs were recruited by lower cost mechanisms compared with 40% of PhD pursuants recruited by higher cost mechanisms. All of the students in our program who pursued STEM professional degrees were recruited by lower cost mechanisms (Figure 2). Thus, in our small sample, lower cost mechanisms are statistically as effective as higher cost mechanisms in recruiting students who transition to PhD, *F*(1, 53) = 1.172, *p* = .284. Using η^2^ as the measure of effect size, cost of recruitment only accounted for 2% of the total variability among those pursuing PhDs.

## Discussion

To our knowledge, this study shows for the first time the cost-effectiveness of recruitment strategies in attracting URM participants to a diversity research summer program who progress to desired outcomes. Few studies have mentioned recruitment mechanisms for summer programs, and only one has indicated the outcome of students who were recruited by certain mechanisms (on- vs. off-campus; Foertsch et al., 1997). Overall, the literature contains little that shows the results of students who were recruited by specific strategies. The findings of this study provide a tool for leaders of diversity research programs to evaluate their current recruitment methods and to incorporate new ones. While we do not owe the outcomes of the participants to these recruitment mechanisms, we do believe this study indicates to program directors that lower cost strategies can be utilized effectively to attract students who are on the path to graduate and professional degrees.

OGR, like other diversity research programs, is devoted to the success of URM in STEM, where students build their skills and are provided with the opportunity to gain research experience and prepare for graduate studies. This study demonstrates a selective, competitive, gender-balanced, URM-centered program, where students were recruited from diverse institutions using a host of mechanisms. The recruitment mechanisms that are the focus of this article are quite common and available for all programs. We have demonstrated how these mechanisms can be assessed to trace their effectiveness to participant outcomes. The five mechanisms of recruitment, email, referrals, event, websites, and other, were not statistically different among non-matriculants and matriculants as single mechanisms. However, a number of statistical differences were evident when we compared the mechanisms and the demographics of the applicants. We note a gender difference in how males and females were recruited to apply to the OGR program. For females, email and events were the most effective and for males, referrals and websites were most effective. As demonstrated in Table 5, each gender was drawn to the program by one low-cost and one high-cost mechanism. These mechanisms can be further classified as high contact (e.g., referrals, events) and low contact (e.g., email, website), which indicates the level of personalization the mechanisms required. For instance, referrals and events required in-person contact or some prior relationships established by the program staff with individual applicants or faculty/administrators at a particular institution, whereas email and websites did not require this personalization or level of contact. Similarly, males and females were attracted to the program by one low-contact and one high-contact mechanism; whereas the high-contact mechanism was most important for males, a low-contact mechanism attracted the most females. It would be tempting to speculate that because of the data noted earlier regarding the low number of minority males compared with their female counterparts, using a high-contact mechanism such as referral may indicate that some advocacy may have played a role on behalf of the referee. More data are needed to make inferences about why this gender difference exists, but it can be assumed that a diverse set of methods is essential to ensure gender balance in the program.

When comparing race for specific mechanisms, differences were noted. Email was most effective for Caucasians, website for Asians, and events for Blacks. These strategies are a mix of high and low cost, but high contact was significant for a portion of the target URM groups, corresponding with data showing events were significant for recruiting students who attended HBCUs. This may verify what is assumed anecdotally when recruiting URM, namely, Blacks/African Americans in this case, that recruiters must search and go where these students are to diversify their programs. This may certainly be true for Black females, who have been noted to select graduate schools based on relationships and environments that appear to be more supportive (Upton & Tanenbaum, 2014).

Outcome data indicate that overall the OGR program was successful: Over 50% of alumni were involved in advanced training in STEM, including 27% enrolled in PhD or MD/PhD programs. In light of this, our data do highlight some interesting points regarding race and ethnicity. Although the percentage of Hispanics and non-Hispanics only differed by 10% in our matriculant pool favoring non-Hispanics, the percentage of Hispanics who matriculated to the PhD was twice that of non-Hispanics (67% vs. 33%). The largest group of non-Hispanics was Blacks, who earned slightly more than half of STEM professional degrees. This would suggest that ethnicity and race even within URM might play a role in matriculation to terminal degrees in STEM. Nationally, both groups are underrepresented in the health professions (e.g., medicine and dentistry), and according to the American Association of Medical Colleges (AAMC; 2014) and the American Dental Association, Blacks and Hispanics make up 9% and 8%, respectively, of working physicians and dentists (Wyckoff, 2010). This is similar to the number reported by Poirier et al. (2009) for STEM PhDs. Additional data indicate that, nationally, Hispanic college enrollment has increased and now outnumbers the enrollment of Blacks in baccalaureate and doctoral degree attainment in STEM. Therefore, this study may be reflective of this national trend (Fry & Lopez, 2012; NSF, 2013).

In this study, we asked what variables were important for underrepresented students in our study to pursue a PhD. We wanted to determine whether the type of recruitment mechanism and the relative cost played a role along with other variables. Using a binary logistic regression analysis, the variables that were strong predictors of students who chose to enroll in PhD programs following their undergraduate training were determined. The following five variables emerged as reliably distinguishing between PhD pursuants and non-PhD pursuants: Carnegie classification, gender, ethnicity, GPA, and relative cost of recruitment mechanism (test of model coefficients: χ^2^ = 12.322, *p* = .03 with *df* = 5; Table 11). Of these five variables, Carnegie classification was the variable that proved to be the strongest positive predictor (*p* = .028), where students attending research universities with high or very high research activity were 5 times more likely to enroll in PhD programs versus those not enrolled in this type of university. While high-cost recruitment mechanisms (events and website) were positive predictors of students pursuing PhDs, they were not statistically significant positive predictors (*p* = .11). Although our data set is quite small, our model corresponds with national data indicating that, overall, most PhD recipients hail from baccalaureate institutions that are research intensive (Fiegener & Proudfoot, 2013). The majority of our PhD pursuants in our sample were Hispanics and, of the HSIs in our data that produced PhD pursuants, 83% were research universities with high or very high activity. However, for Black students from HBCUs who pursued PhDs, their schools were not classified as research universities and the same for those pursuing STEM professional degrees, which may also account for our model results, where HBCUs regardless of research intensity have been noted to play a major role in Blacks pursuing PhDs (Bonner, Alfred, Lewis, Nave, & Frizell, 2009). With this correspondence to national data, we believe that a more robust examination of the relative cost of recruitment mechanism may provide us with clearer evidence of its contribution to the pursuit of the PhD pathway.

### Limitations and Implications for Future Studies

There are several limitations to this study. As indicated above, one limitation is the sample size and the focus on one program, which may have prevented statistical significance in some of our data as well as determining the level of contribution of factors to the PhD path within our model. In examining our recruitment mechanisms, we disaggregated data from an already small sample size; thus, our data are suggestive rather than conclusive, regarding the effectiveness of our mechanisms. Also, this study lacks outcome data for non-matriculants, which would provide definition to our recruitment mechanisms and their association with outcomes. As our program is ongoing, we continue to collect these data, which may increase our sample size for future studies. We can examine other programs with similar foci controlling for variation in program design to validate our findings. We believe that our data provide a skeleton for a larger study to examine recruitment mechanisms in detail for summer research programs that can also be translated to other programs (e.g., graduate programs, postbaccalaureate programs).

These data should not be considered in isolation. We acknowledge that there are many factors involved in the path to the PhD for all students and those in our programs. In no way are we suggesting that our mechanisms are responsible for students pursuing STEM doctorates or professional degrees. Factors such as the quality of the research experiences during the OGR program, prior- and post-OGR research experience, and quality of their application to PhD programs are just among the few factors responsible for entry into graduate and professional programs, which we did not consider here, but may be interesting to add to a more comprehensive study. In speaking of recruitment only, however, it might be interesting to note the “dosage” of recruitment and the relationship development to recruit a student and then look at the outcome. In other words, using the mechanisms we outlined, does the number of times an individual student was contacted prior to matriculation into a program matter to the outcome? Currently, the number of times individuals were contacted by our program, from initial contact to application, is recorded, but the amount of contact from application to matriculation is not captured, so the focus of this study was the front-end recruitment.

We know that selection matters to the outcomes we observed, whether it is self-selection by the student to apply to our program or selection of matriculants by program leadership. Our program sought students interested in research; thus, some students were on track to the PhD when they entered OGR. However, in our selection of participants, GPA, prior research experience, and specific coursework were not the major determinants of selection. We certainly accepted students who were conducting research for the first time or deciding on a career path. A more in-depth study is needed to determine the true effect of OGR on the outcomes.

Another limitation may be that we have not put into practice newer and possibly more innovative methods of recruitment (e.g., social media and virtual recruitment fairs) to determine if they are effective in bringing students to our program and in attracting students who pursue terminal degrees in STEM. While we recently implemented webinars to provide information on OGR, the email mechanism was still the initial mode of contact to attract participants to the webinar.

## Conclusion

This study demonstrates for the first time that cost-effective measures can be utilized to recruit students who will ultimately pursue PhDs or STEM professional degrees. We provide evidence that establishing a system to implement lower cost mechanisms may be worth an investment of time for individual programs, especially during fiscally lean times. Higher cost mechanisms, namely, conferences, are recruitment staples and have great value, which according to our data, part of their value is in recruiting Blacks and students from HBCUs. But we also show the value of lower cost mechanisms, and we suggest that this value is that using these mechanisms does not compromise program outcomes, while conserving program dollars. We believe that lower cost recruitment mechanisms can be used to supplement higher cost ones and should be given great consideration in program recruitment plans. We also advocate that summer programs designed to attract URM students thoroughly track the recruitment mechanisms and the outcomes by the mechanisms because this may be a key to improved efficiency.

We caution that our data must be viewed as a part of the overall experience for the student. As a whole, we are not suggesting that our program is solely responsible for the outcomes, rather our program was a part of the students’ experiences and there were many factors that contributed to the outcomes, those within and outside of the OGR program goals. We also want to make clear that by recruiting students via certain mechanisms we are not guaranteeing a particular outcome nor are we implying the mechanism is responsible for the outcome. What we do suggest is that by assessing mechanisms comprehensively in light of small recruitment budgets, cost-effective mechanisms may be utilized, and in our program, these mechanisms were successful in attracting URM students who pursued terminal degrees in STEM.

## Figures and Tables

**Table 1.** Description of OGR Recruitment Mechanisms.

Mechanism	Description
Emails	Recruitment emails and electronic flyers with program information were sent to chairs of STEM departments at MSIs, institutions ranked by periodicals as top producers of URM graduates in STEM (e.g., Diverse issues of Higher education, Hispanic outlook, Winds of Change), and program directors of MARC-USTAR, MBRS-RISE, and LSAMP
Referrals	Word of mouth by colleagues of program staff, other faculty, and OGR students; shared applications from other DAP programs and other on-campus summer programs
Events	Program staff attended large URM student focused conferences (e.g., ABRCMS, SACNAS) and smaller internship and campus career fairs at MSIs and visited individual classes and program meetings at MSIs and non-MSIs.
Websites	Includes the OGR webpage and other websites across the country. The URL for the OGR website was included on all OGR media (flyers, ink pens, emails, etc.); the URL was posted on the career/internship/program websites at various institutions and appeared on national webpages (e.g., Institute for Broadening Participation and NHGRI)
Other	Ads and fliers were generated and mailed to institutions (all types) within the Missouri/Illinois area and to some MSIs that border these states. This also includes when no indication was given how a student heard about the program or if we could not verify against our database the method listed by an applicant

*Note*. OGR = Opportunities in Genomics Research; STEM = science, technology, engineering, and mathematics; MSI = minority-serving institution; URM = underrepresented minorities; MARC-USTAR = Maximizing Access to Research Careers–Undergraduate Student Training in Academic Research; MBRS-RISE = Minority Biomedical Research Support–Research initiative for scientific enhancement; LSAMP = Louis Stokes Alliance for Minority Participation; DAP = Diversity Action Plan; URM = ABRCMS = Annual Biomedical Research Conference for Minority Students; SACNAS = Society for the Advancement of Chicanos and Native Americans in Science; URL = uniform resource locator; NHGRI = National Human Genome Research Institute.

**Table 2.** Demographics for OGR Applicants Disaggregated by Matriculation Status.

	Non-matriculants (*n* = 126)		Matriculants (*n* = 59)	
	*N*	Percent	*N*	Percent
Gender				
Male	43	34.1%	27	45.8%
Female	83	65.9%	32	54.2%
Ethnicity				
Hispanics	32	25.4%	26	44.1%^†^
Non-Hispanics	88	69.8%	33	55.9%
Race				
Asian	10	7.9%	2	3.4%
Black/African American	52	41.3%	29	49.2%
Caucasian	27^†^	21.4%	3	5.1%
Multiracial	7	5.6%	1	1.7%
Native American	1	.8%	1	1.7%
No response	29	23.0%	23	39.0%

*Note. N* = number of applications.

† Statistical significance, *p* ≤ .05.

**Table 3.** Institution Classifications for OGR Applicants Disaggregated by Matriculation Status.

	Non-matriculants (*n* = 126)		Matriculants (*n* = 59)	
	*N*	Percent	*N*	Percent
MSI classification				
HBCU	33	26.2%	18	30.5%
HSI	25	19.8%	19	32.2%
Other MSI	5	4.0%	0	0.0%
Non-MSI	63	50.0%	22	37.3%
Carnegie classification				
Associates	3	2.4%	1	1.7%
Baccalaureate	31	24.6%	16	27.1%
Master’s	35	27.8%	10	16.9%
Doctoral	8	6.3%	6	10.2%
Research (high/very high)	49	38.9%	26	44.1%

*Note. N* = number of applications. OGR = Opportunities in Genomics Research; MSI = minority-serving institution; HBCU = Historically Black College and University; HSI = Hispanic Serving Institution.

**Table 4.** Recruitment Mechanisms for OGR Applicants Disaggregated by Matriculation Status.

Recruitment mechanism	Non-matriculants (*n* = 126)		Matriculants (*n* = 59)	
	*N*	Percent	*N*	Percent
Email	45	35.7	19	32.2
Event	27	21.4	14	23.7
Referral	38	30.2	22	37.3
Website	13	10.3	3	5.1
Other	3	2.4	1	1.7

*Note. N* = number of applications.

**Table 5.** Demographics for OGR Applicants Disaggregated by Recruitment Mechanism.

	Email		Event		Referral		Website		Other	
	*N*	%	*N*	%	*N*	%	*N*	%	*N*	%
Gender										
Male	18	25.7	7	10.0	31^†^	44.3	11^†^	15.7	3	4.3
Female	46^†^	40.0	34^†^	29.6	29	25.2	5	4.3	1	0.9
Ethnicity										
Non-Hispanic	40	33.1	30	24.8	38	31.4	11	9.1	2	1.7
Hispanic	23	39.7	10	17.2	21	36.2	2	3.4	2	3.4
Race										
Asian	3	25.0	1	8.3	4	33.3	4^†^	33.3	0	0.0
Black/African American	25	30.9	25^†^	30.9	29	35.8	2	2.5	0	0.0
Caucasian	15^†^	50.0	1	3.3	8	26.7	4	13.3	2	6.7
Multiracial	2	25.0	3	37.5	2	25.0	0	0.0	1	12.5
Native American	1	50.0	0	0.0	0	0.0	0	0.0	1^†^	50.0
No response	18	34.6	11	21.2	17	32.7	6	11.5	0	0.0

*Note. N* = number of applications.

† Statistically significant, *p* ≤ .05

**Table 6.** Institution Classifications for OGR Applicants Disaggregated by Recruitment Mechanism.

	Email		Event		Referral		Website		Other	
	*N*	%	*N*	%	*N*	%	*N*	%	*N*	%
MSI classification										
Non-MSI	23	27.1	13	15.3	34	40.0	13^†^	15.3	2	2.4
HBCU	18	35.3	21^†^	41.2	11	21.6	1	2.0	0	0.0
HSI	22	50.0	5	11.4	13	29.5	2	4.5	2	4.5
Other MSI	1	20.0	2	40.0	2	40.0	0	0.0	0	0.0
Carnegie classification										
Associates	0	0.0	1	25.0	2	50.0	1	25.0	0	0.0
Baccalaureate	13	27.7	12	25.5	18	38.3	2	4.3	2	4.3
Master’s	21	46.7	13	28.9	7	15.6	3	6.7	1	2.2
Doctoral	6	42.9	1	7.1	5	35.7	2	14.3	0	0.0
Research (high/very high)	24	32.0	14	18.7	28	37.3	8	10.7	1	1.3

*Note. N* = number of applications. OGR = Opportunities in Genomics Research; MSI = minority-serving institution; HBCU = Historically Black College and University; HSI = Hispanic Serving Institution.

† Statistically significant, *p* ≤ .05

**Table 7.** Current Career Outcomes for Students Completing OGR Program Between 2007 and 2013.

	*N*	%
Other	25	45.5
Postbacc/master’s program	6	10.9
STEM professional degree	9	16.4
PhD degree	15	27.3

*Note. N* = number of OGR alumni pursuing a specific career path. OGR = Opportunities in Genomics Research.

**Table 8.** Demographics for Students Completing OGR Program Disaggregated by Career Outcomes.

	PhD		STEM professional degree		Master’s/postbacc		Other	
	*N*	%	*N*	%	*N*	%	*N*	%
Gender								
Male	8	53.3	3	33.3	3	50.0	12	48.0
Female	7	46.7	6	66.7	3	50.0	13	52.0
Ethnicity								
Non-Hispanic	5	33.3	5	55.6	5	83.3	15	60.0
Hispanic	10	66.7	4	44.4	1	16.7	10	40.0
Race								
Asian	0	0.0	0	0.0	0	0.0	1	4.0
Black/African American	4	26.7	5	55.6	4	66.7	14	56.0
Caucasian	1	6.7	0	0.0	1	16.7	1	4.0
Multiracial	0	0.0	0	0.0	0	0.0	1	4.0
Native American	1	6.7	0	0.0	0	0.0	0	0.0
No response	9	60.0	4	44.4	1	16.7	8	32.0

*Note. N* = number of OGR alumni pursuing a specific path by demographic. OGR = Opportunities in Genomics Research; STEM = science, technology, engineering, and mathematics; MSI = minority-serving institution.

**Table 9.** Institution Classification for Students Completing OGR Program Disaggregated by Career Outcomes.

	PhD		STEM professional degree		Master’s/postbacc		Other	
	*N*	%	*N*	%	*N*	%	*N*	%
MSI								
Non-MSI	7	46.7	3	33.3	2	33.3	8	32.0
HBCU	2	13.3	3	33.3	3	50.0	9	36.0
HSI	6	40.0	3	33.3	1	16.7	8	32.0
Other MSI	0	0.0	0	0.0	0	0.0	0	0.0
Carnegie								
Associates	0	0.0	0	0.0	0	0.0	1	4.0
Baccalaureate	4	26.7	1	11.1	3	50.0	7	28.0
Master’s	0	0.0	2	22.2	0	0.0	7	28.0
Doctoral	0	0.0	1	11.1	1	16.7	4	16.0
Research (high/very high)	11^†^	73.3	5	55.6	2	33.3	6	24.0

*Note. N* = number of OGR alumni pursuing a specific path by institution type. OGR = Opportunities in Genomics Research; STEM = science, technology, engineering, and mathematics; MSI = minority-serving institution; HBCU = Historically Black College and University; HSI = Hispanic Serving Institution.

† Statistically significant, *p* ≤ .05.

**Table 10.** Recruitment Mechanism for Students Completing OGR Program Disaggregated by Career Outcomes.

	PhD		STEM professional degree		Master’s/postbacc		Other	
Mechanism	*N*	%	*N*	%	*N*	%	*N*	%
Email	4	26.7%	4	44.4%	1	16.7%	8	32.0%
Event	6	40.0%	0	0.0%	2	33.3%	5	20.0%
Referral	4	26.7%	5	55.6%	2	33.3%	10	40.0%
Website	0	0.0%	0	0.0%	1	16.7%	2	8.0%
Other	1	6.7%	0	0.0%	0	0.0%	0	0.0%

*Note. F*(4, 50) = .858, *p* = .496; observed power = .254, η^2^ = 0.06. *N* = number of OGR alumni pursuing a specific path by mechanism. OGR = Opportunities in Genomics Research; STEM = science, technology, engineering, and mathematics.

**Table 11.** Logistic Regression Modeling of Factors Predicting Pursuit of PhD (*n* = 55).

	Unstandardized coefficients		Odds ratio [95% CI]
	*B*	*SE B*	Exp(*B*)
Gender (female)	−0.64	0.72	0.53 [0.13, 2.17]
GPA	−0.23	0.71	0.80 [0.20, 3.21]
Ethnicity (Hispanic)	1.11	0.75	3.04 [0.69, 13.31]
Recruitment costs (high)	1.25	0.79	3.48 [0.74, 16.29]
Carnegie classification (high/very high research)	1.62	0.75	5.07 [1.16, 22.10]*

Note. Nagelkerke *R*^2^ = .291. CI = confidence interval; GPA = grade point average.

* *p* < .05.
